# Geographic variation in the matching between call characteristics and tympanic sensitivity in the Weeping lizard

**DOI:** 10.1002/ece3.8469

**Published:** 2021-12-14

**Authors:** Antonieta Labra, Claudio Reyes‐Olivares, Felipe N. Moreno‐Gómez, Nelson A. Velásquez, Mario Penna, Paul H. Delano, Peter M. Narins

**Affiliations:** ^1^ Department of Biosciences Centre for Ecological and Evolutionary Synthesis (CEES) University of Oslo Oslo Norway; ^2^ Programa de Fisiología y Biofísica Instituto de Ciencias Biomédicas Facultad de Medicina Universidad de Chile Santiago de Chile Chile; ^3^ Departamento de Biología y Química Facultad de Ciencias Básicas Universidad Católica del Maule Talca Chile; ^4^ Departamento de Neurociencia Facultad de Medicina Universidad de Chile Santiago Chile; ^5^ Centro Avanzado de Ingeniería Eléctrica y Electrónica AC3E Universidad Técnica Federico Santa María Valparaíso Chile; ^6^ Department of Integrative Biology & Physiology University of California Los Angeles Los Angeles California USA

**Keywords:** eardrum, laser Doppler vibrometry, *Liolaemus chiliensis*, peripheral auditory sensitivity, tympanic membrane, ultrasound

## Abstract

Effective communication requires a match among signal characteristics, environmental conditions, and receptor tuning and decoding. The degree of matching, however, can vary, among others due to different selective pressures affecting the communication components. For evolutionary novelties, strong selective pressures are likely to act upon the signal and receptor to promote a tight match among them. We test this prediction by exploring the coupling between the acoustic signals and auditory sensitivity in *Liolaemus chiliensis*, the Weeping lizard, the only one of more than 285 *Liolaemus* species that vocalizes. Individuals emit distress calls that convey information of predation risk to conspecifics, which may respond with antipredator behaviors upon hearing calls. Specifically, we explored the match between spectral characteristics of the distress calls and the tympanic sensitivities of two populations separated by more than 700 km, for which previous data suggested variation in their distress calls. We found that populations differed in signal and receptor characteristics and that this signal variation was explained by population differences in body size. No precise match occurred between the communication components studied, and populations differed in the degree of such correspondence. We suggest that this difference in matching between populations relates to evolutionary processes affecting the Weeping lizard distress calls.

## Graphical abstract

We compared the matching between signals (distress calls) and reception (tympanic sensitivities) in two distant located populations of the lizard *Liolaemus chiliensis*. Populations differed in both characteristics and there was no support for a complete match between the studied communication components. Moreover, populations differed in their degree of matching.

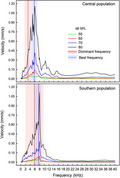

## 1 INTRODUCTION

Communication is a multi‐component process essential for the interactions between sender and receiver (Hauser, 1997; Labra, 2020). The effectiveness of this process depends on the extent of matching between signal production and transmission by the sender and the signal reception and decoding by the receiver. In addition, this process requires signal adaptation to the environmental conditions over which the signal propagates (Bradbury & Vehrencamp, 2011; Endler, 1993; Gerhardt, 1994). Thus, multiple factors can affect the communication between sender and receiver (Bradbury & Vehrencamp, 2011; Endler, 1993; Narins & Zelick, 1988). Data show, however, that the associated costs of the communication process—for example, energy use in signaling, increase in predation risk (Outomuro et al., 2017; Ryan, 1988; Vehrencamp et al., 1989; Zahavi & Zahavi, 1999; Zhao et al., 2016)—may act as selective pressures to promote the coevolution of these components, thus ensuring effective communication (Endler, 1992, 1993). Presently, an extensive literature documents coevolution among the communication components at macro‐and micro‐evolutionary scales, involving different sensory modalities and taxa (e.g., Alberts, 1992; Brand et al., 2020; Cobo‐Cuan & Narins, 2020; Driessens et al., 2017; Grace & Shaw, 2011; Lall et al., 2010; Ng et al., 2013; Price, 2017; Sato & Sorensen, 2018; Sheehan et al., 2014).

Acoustic communication has been an important target to explore the coevolution and matching among communication components (e.g., Charlton et al., 2019; Manley & Kraus, 2010). However, some studies have also revealed that these components may show only a partial matching (e.g., Gerhardt & Schwartz, 2001; Kostarakos et al., 2009; Meenderink et al., 2010), as in the case of the advertisement calls emitted by the frog *Pleurodema thaul*, which are poorly adapted to the local sound degradation across the species distribution (Velásquez et al., 2018). In addition, only males exhibit strong responses to calls from their population compared to those of other populations (i.e., local vs. non‐local calls; Velásquez et al., 2014, 2015). Furthermore, other studies have revealed complete uncoupling of the communication components. For example, two *Brachycephalus* frog species that lost their capability to hear their calls have retained the ability to vocalize (Goutte et al., 2017). Some factors that might explain this partial or total uncoupling include a short time for the selective pressures to act upon the paired adaptation of these components, the action of stochastic forces having stronger effects than the selective pressures that modulate the communication processes (e.g., Irwin et al., 2008; Kostarakos et al., 2009), and/or different selective pressures or evolutionary rates of the components (Ballentine, 2006; Betancourth‐Cundar et al., 2016; Zhao et al., 2016).

A singular opportunity to explore the evolution of the matching among the communication components is provided by an evolutionary novelty, that is, “a new feature (structure or behavior) in a group of organisms (taxa) that is not homologous to a feature in an ancestral linage” (Hall & Kerney, 2012) or related taxa (Davis, 2012). Such is the case of the occurrence of ultrasonic vocalizations in two phylogenetically unrelated frog species (Arch & Narins, 2008), the "vocal cords" in the snake species, *Pituophis melanoleucus* (Young et al., 1995), or the rattle of rattlesnakes (Allf et al., 2016). For a functional novelty involved in communication, that is, not just a by‐product of unrelated processes, its components likely show a high degree of coupling, modulated by strong selective pressures. We tested this hypothesis by studying the matching between the characteristics of the vocalizations emitted by the Weeping lizard, *Liolaemus chiliensis* (Labra et al., 2013), and its peripheral auditory sensitivity in two populations widely separated (>700 km) throughout its latitudinal distributional range.

The vocalizations of the Weeping lizard can be considered an evolutionary novelty among *Liolaemus* because this is the only species known to vocalize within one of the most speciose lizard genera in the world (>285 spp; Uetz & Hošek, 2021). The occurrence of vocalizations in three distantly related *Liolaemus* species has been anecdotally reported (for a review see Reyes‐Olivares & Labra, 2017), but more recent data do not support sound production for at least two of these three species (Reyes‐Olivares, personal observation). Lizards vocalize when seized by a predator (i.e., distress calls; Labra et al., 2013), and these calls act as warning signals of predation risk since conspecifics reduce their activity upon hearing the calls (Hoare & Labra, 2013; Labra et al., 2016; Ruiz‐Monachesi & Labra, 2020). Immobility may enhance the likelihood that individuals remain undetected by nearby predators, as prey detection usually depends on the target movement (Magellan, 2019). Furthermore, calls may also convey information about the level of predation risk, which is decoded by the lizards responding accordingly to the threat (Ruiz‐Monachesi & Labra, 2020). Therefore, these distress calls are functional vocalizations, typically eliciting conspecific reactions. Moreover, the structure of these calls, that is, harmonics and nonlinear phenomena (Labra et al., 2013), suggests that vocal structures that modulate air pressure have evolved concomitantly (Fitch et al., 2002; Russell & Bauer, 2020).

The distress calls of the Weeping lizard seem to show geographic differences (Labra et al., 2016; Pincheira‐Donoso & Núñez, 2005), and therefore, our first aim was to characterize this variation. Furthermore, as selective pressures likely promote the matching of the communication components to ensure the functionality of this evolutionary novelty, we predict that these populations will have peripheral auditory (i.e., tympanic) sensitivities matching their respective call characteristics. As such, both populations should show similar matching between vocal and auditory components.

## 2 MATERIALS AND METHODS

### 2.1 Lizards and maintenance

**FIGURE 1:**
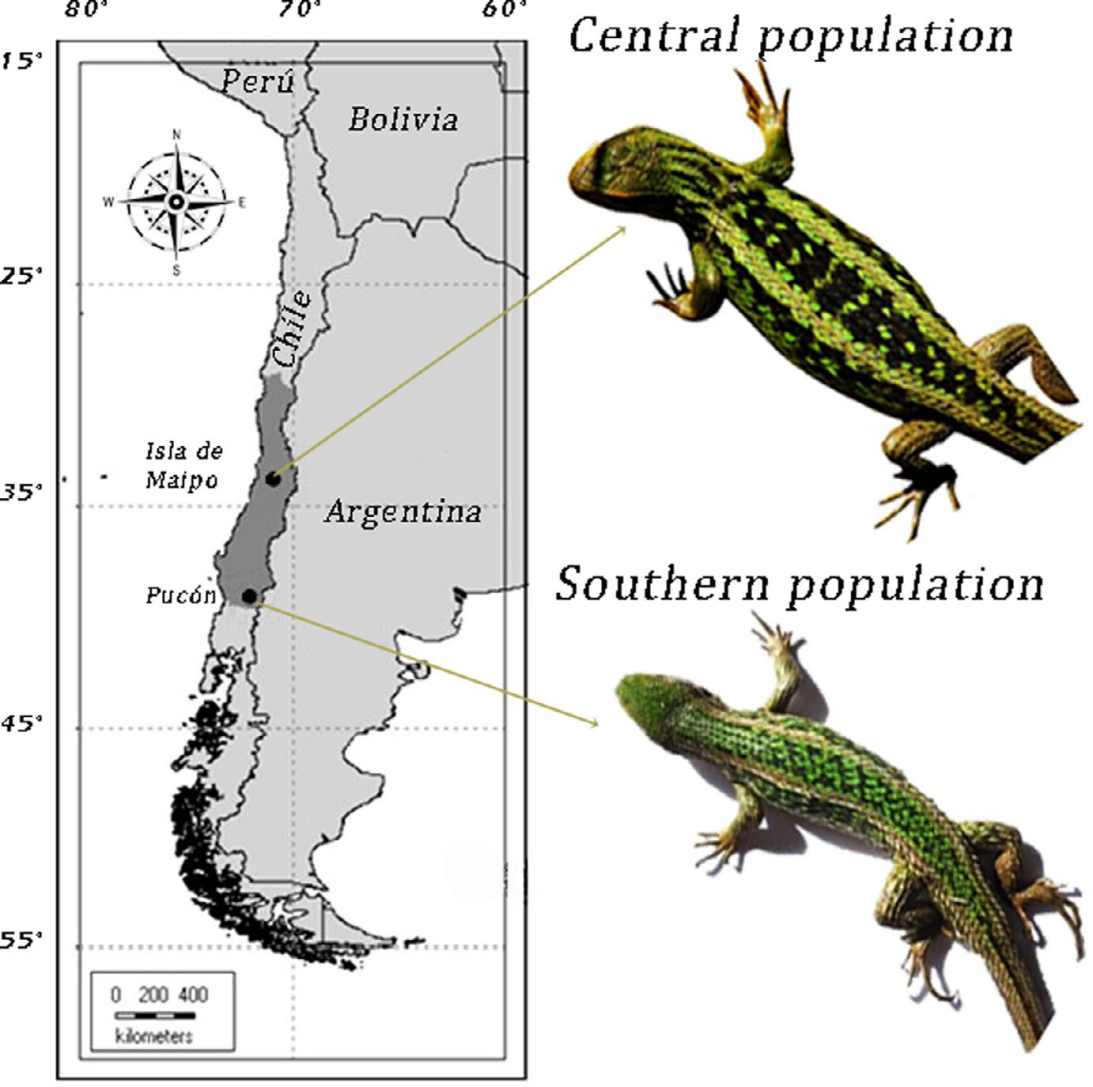
The geographical distribution of *Liolaemus chiliensis* in Chile is indicated by the dark gray area (Demangel, 2016). Locations of the studied populations and a picture of a typical individual from each population

*Liolaemus chiliensis*, an iguanid lizard endemic to Chile and Argentina, inhabits sclerophyllous and xerophilous scrublands across a wide latitudinal range (~1000 km in Chile; Demangel, 2016). We collected adults from two populations (Figure 1): (a) Isla de Maipo, in the central area of the species distributional range (33°44′S, 70°55′W; henceforth: central population), and (b) Pucón, at the southernmost extent of the species distribution (39°16′S, 71°58′W; henceforth: southern population). Previous studies on distress calls of *L*. *chiliensis* included a Melipilla population (Labra et al., 2013, 2016), which was no longer available to be studied. Therefore, we collected individuals from the nearby population, Isla de Maipo. We transported animals to the laboratory, where we maintained them following Hoare and Labra (2013). Briefly, lizards were housed individually in plastic enclosures (44.5 × 32 × 25 cm), kept in an indoor vivarium with continuous ventilation, with temperatures ranging between 33 and 12°C and a 13:11 light–dark cycle. We fed lizards with mealworms dusted with vitamins for reptiles (SERA reptimineral C). Individuals remained undisturbed (except for feeding) for 2 days or a week before the vocal and tympanic sensitivity recordings, respectively. Following the experiments, lizards were released in healthy condition at their georeferenced collecting points.

### 2.2 Distress call recordings

During the austral spring–summer of 2011–2013, we collected 14 lizards from the central population (7 ♀ and 7 ♂; mean snout‐vent length (SVL): 86.3 ± 2.13 standard error (*SE*) mm) and 26 (13 ♀ and 13 ♂; SVL: 70.5 ± 1.10 *SE* mm) from the southern population. Following Labra et al. (2013), we recorded the vocalizations between 11:00 and 16:00 h in a sound‐attenuated booth in which walls and the ceiling were covered with 50‐cm‐height foam wedges. Before a recording, and to avoid variations in body temperature that could affect sound production (e.g., Crowley & Pietruszka, 1983), lizards were exposed to a heat source to allow them to thermoregulate and achieve the species preferred value (~35°C; Labra et al., 2009). After vocal recordings, we measured the cloacal temperatures and excluded vocalizations from individuals with temperatures beyond 35 ± 2°C (mean ± *SE*). Additional recordings from a given individual were obtained after a minimum of 48 h. We evoked distress calls by gently grasping the lizard with the thumb and forefinger, and softly touching its snout with a finger for 2 min (Labra et al., 2013). The lizard was positioned 10 cm in front of a directional microphone (Sennheiser ME 66; frequency response: 40–22 kHz) connected to a digital recorder (Tascam DR‐100). For the southern recordings, we also obtained the sound levels (in dB SPL) by positioning at 10 cm in front of the focal lizard, a precision integrating sound level meter (Brüel & Kjær 2230), previously calibrated with a sound level calibrator (Brüel & Kjær 4230); the SPL values were dictated to the recorder (Labra et al., 2007). For each individual, we averaged all its recorded sound levels independently of the emitted call type (see below). The generated.WAV files (44.1 kHz, 16 bits) were high‐pass filtered (cutoff: 200 Hz) and analyzed using Raven Pro 1.3 (Cornell Laboratory of Ornithology, Ithaca, NY).

**FIGURE 2:**
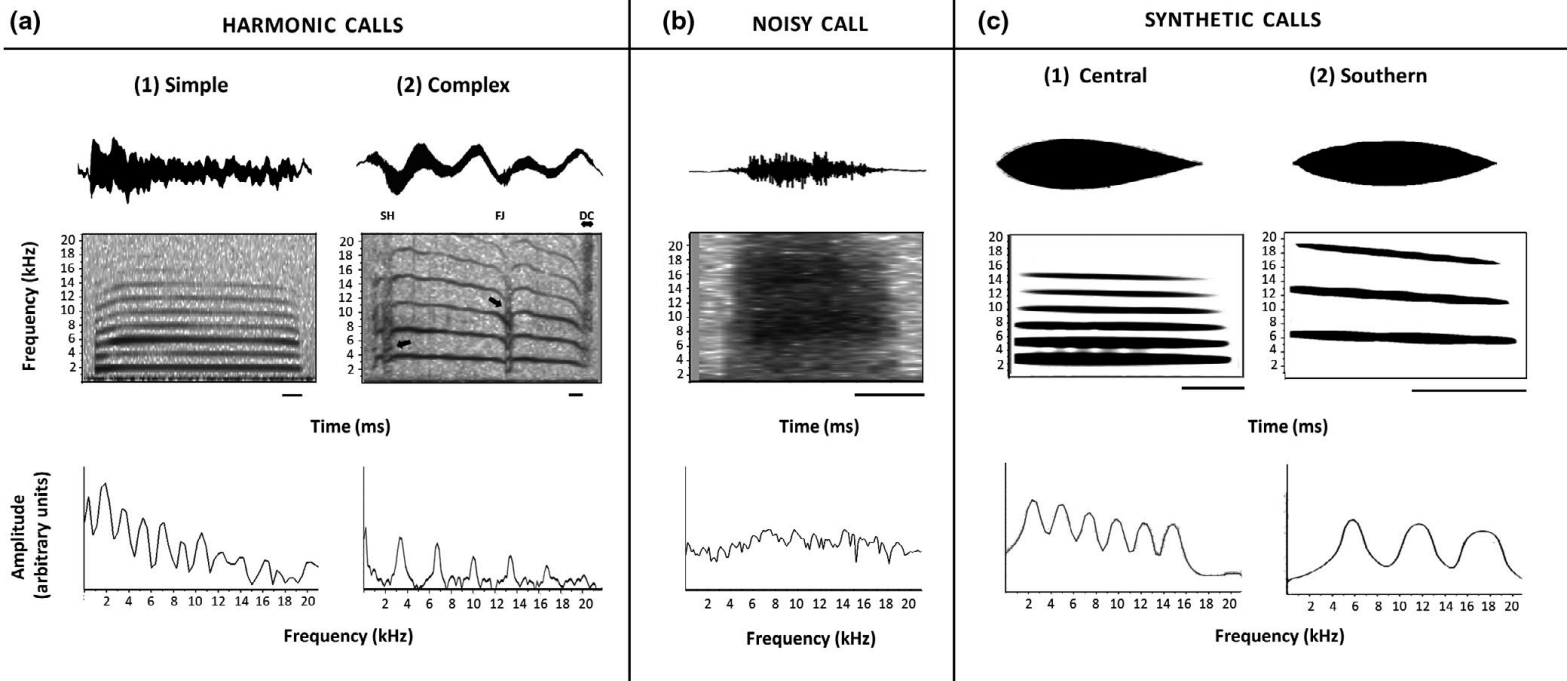
Oscillograms, sound spectrograms, and power spectra of the distress calls of *Liolaemus chiliensis*. The amplitude axis is not comparable among plots. (a) Harmonic calls. Two call types are shown, simple and complex, without and with nonlinear phenomena (Labra et al., 2013), respectively. The complex call contains three nonlinear phenomena (indicated by the arrows and letters on top), which from left to right are: (i) subharmonics (SH), (ii) frequency jump (FJ), and (iii) deterministic chaos (DC). The harmonic calls, simple and complex, were emitted by a female (snout‐vent length ‐SVL‐ = 90.7 mm) and a male (SVL = 69.6 mm), respectively, from the central population. (b) A noisy call emitted by a male (SVL = 62.9 mm) from the southern population. (c) The synthetic calls of each population used to determine their tympanic responses. Scale bars in each panel: 20 ms

By visual inspection (e.g., Eckenweber & Knörnschild, 2016), we identified two types of distress calls: (a) harmonic (Figure 2a): calls with a complete or partial clear harmonic structure, and (b) noisy or non‐harmonic (Figure 2b): calls not having any clear harmonic structure (i.e., turbulent noise; Fitch et al., 2002). We further classified harmonic calls as simple or complex (Figure 2a), based on the absence or presence of nonlinear phenomena, respectively (Labra et al., 2013). We measured the duration (ms) of all call types, and for the harmonic calls, we also determined the number of harmonics recognizable in the spectrograms (fast Fourier transform length = 1024, Hamming Window = 87.5% overlap, resolution: frequency = 488 Hz; time = 0.256 ms), as this variable may modulate the responses to distress calls (Aubin & Bremond, 1992) and might help to discriminate between populations (e.g., Eiler & Banack, 2004). In addition, from the oscillograms, we also obtained the time to the maximum amplitude (ms) measured from the start of the call, while from the fast Fourier transform, we obtained the fundamental and dominant frequencies. These frequencies, and the number of harmonics, were measured in a segment free from nonlinear phenomena, preferably at the beginning of the signal (Labra et al., 2013). Although calls of this lizard species contain ultrasonic components (Labra et al., 2013), we did not detect them, as our microphone was nominally sensitive up to 22 kHz. However, since the energy in these calls decreases gradually toward the higher frequencies without energy gaps (Labra et al., 2013), we considered that calls with frequencies between 20 and 22 kHz contained ultrasonic components, which provides an estimate of the occurrence of ultrasound in these calls.

### 2.3 Tympanic membrane recordings

We collected new individuals during the austral summer of 2014 (December)–2015 (January); 13 (7♀, 6♂; SVL 84.60 ± 2.22 mm) and 11 lizards (7 ♀, 4 ♂; 71.62 ± 3.89 mm) from the central and southern populations, respectively. Before a recording, the focal lizard was lightly anesthetized (i.e., motionless, but with normal lung respiration) via an intramuscular injection of Virbac Zoletil^®^ 50 (0.4 µl/g body mass) in a forearm. This dosage was typically effective for 2–3 h, though some individuals required an additional half dose to complete the recordings. Experiments were done in the sound‐attenuated booth previously described, where the anesthetized lizard was placed on a temperature‐controlled (~35°C) thermal plate (ReptiTherm^®^) located on an anti‐vibration table (TMC 63‐500). The response of the left eardrum, or tympanic membrane, to various acoustic stimuli, was measured with a laser Doppler vibrometer (Polytec CLV‐2534). The compact sensor head of the laser was positioned 30 cm from the lizard's eardrum, and the laser beam was aimed perpendicular to the tympanic surface, aimed at the tip of the extracolumellar attachment, close to the center of the eardrum (Christensen‐Dalsgaard & Manley, 2008). We enhanced beam reflection by placing a ~1 mm^2^ flake of highly reflecting white correction tape at the target point of the laser beam (Christensen‐Dalsgaard & Manley, 2005) with the aid of a binocular light microscope (PZO OP‐1, PZO, Warsaw, Poland). The vibrometer sensitivity was set at 5 mm/s, and the incoming signal was amplified by 20 dB with a custom‐made amplifier. Automated custom software recorded the vibrometer output signal and controlled stimulus generation and production. For this, we used a data acquisition card (National Instruments NI‐6071E), a programmable attenuator (PA5, System 3, Tucker‐Davis Technologies, Alachua, FL, USA), and an amplifier (SKP Pro Audio Max 710X). Acoustic stimuli were broadcast for frequencies up to 20 kHz and above this limit, using an audio loudspeaker (Dynaudio BM 6, Skanderborg, Denmark) and an ultrasonic loudspeaker (Fostex Company, Tokyo, Japan), respectively, both placed at 50 cm in front of the focal lizard. We measured the response to ultrasonic frequencies to explore whether these frequencies would be involved in the species communication.

Before the recordings, we calibrated the sound pressure using an ultrasonic ¼” free‐field microphone (GRAS 40BE) powered by a preamplifier (GRAS 26CB), placed above the head of a realistic silicone lizard model (~5 cm), positioned where the focal lizard would be placed later. The GRAS microphone was calibrated within the audible frequency range with a sound level meter (Brüel & Kjær 2238) by broadcasting pure tones of the same frequencies that were used later in the trials. The microphone output was stored, and the SPL obtained was used to automatically adjust the programmable attenuator to the SPL to be used during the recordings. Stimulus generation and signal acquisition were performed at a 200 kHz sample rate using 16‐bit resolution.

All lizards were exposed individually to stimuli consisting of pure tones and synthetic distress calls of each population; for logistic reasons, however, only a subset of eight adults (4 ♀, 4 ♂; SVL 86.61 ± 2.59 mm) from the central population was analyzed for the response to distress calls. We synthesized tones with a custom program, and their duration was 100 ms, with rise and fall ramps of 10 ms. A sequence of tones was presented, starting at 0.1 kHz, and in frequency steps of 0.2 kHz from 0.2 to 9.0 kHz. Then, from 9.0 to 20 kHz and 20 to 40 kHz, the frequency steps were 0.50 and 2 kHz, respectively. After each tone, there was a period of silence of the same duration as the tone. We controlled the intrapopulation variation in the call characteristics by creating one call for each population using Adobe Audition 3.0 (Adobe System Inc.), based on the average spectro‐temporal characteristics of each population harmonic calls (e.g., Fong et al., 2021; Hoare & Labra, 2013). The synthetic calls had a downward frequency modulation pattern, the most frequently found in these populations (see Results). Images of these calls are shown in Figure 2c, and the values of the variables for the call of the central and southern population are, respectively: number of harmonics: six and three, duration: 71 and 42 ms, time to maximum amplitude: 26 and 19 ms, fundamental frequency (which was also the dominant frequency): 2.7 and 6.3 kHz, and a downward sweep from 2.7 to 2.1 kHz and from 6.3 to 5.6 kHz.

Acoustic stimuli were broadcast at 55, 60, 70, and 80 dB SPL. The order of presentation of the stimulus types and the sound levels followed a counterbalanced design to avoid potential effects of order presentation. The signal output of the laser was obtained simultaneously with the stimulus presentation. The acquisition window included the stimulus and its silence interval. For each acoustic stimulus, we recorded 20 response replicates.

The acquired signals were analyzed with a custom‐made script in the R environment (R Core Team, 2020), using the Seewave package (Sueur et al., 2008). For each of the 20 response replicates by tone, we obtained the RMS (root‐mean‐square) of a segment of 80 ms in the middle of the stimulus and in the silence period. We determined the ratio between these RMS values, discarding the values in the first quartile, that is, those with the lowest signal‐to‐noise ratio, to reduce the noise and obtain better responses. The remaining replicates were averaged for further analysis. A fast Fourier transform (window length = 8192 points; frequency resolution = 24.41 Hz) was applied at the mid‐point of the average response to obtain the vibration velocity of the eardrum. Subsequently, we used these measurements to get the velocity transfer function for the different frequencies and sound levels. From these curves, we obtained the maximum velocity and the frequency at which it was measured, that is, the best frequency. Additionally, we characterized the sensitivity of the tympanic response by considering the: (a) sensitivity range: the frequency range over which the eardrum vibrated at least at half of the velocity recorded at the best frequency, and (b) the lower and upper frequency limits of this range.

To analyze the matching between signals and tympanic sensitivities, we recorded the tympanic response to the synthetic distress calls, obtaining the RMS of 20 replicates by call. The values that fell in the first quartile were discarded, and the remaining values were averaged for further analyses. In contrast to the tone analyses, in this case, we used the RMS of the whole stimulus because it showed different temporal characteristics. Mean power spectra of the synthetic distress calls and the tympanic response were obtained with a fast Fourier transform (window length = 2048 points; frequency resolution = 97.66 Hz). This lower frequency resolution, as compared to the one used in the tone analyses, allowed smoother spectra. Finally, we estimated the matching between the spectra of the synthetic distress calls and the tympanic sensitivities, following a method similar to the one used by Moreno‐Gómez et al. (2013), acquiring the spectral cross‐correlations at zero‐lag between these spectra using the function “ccf” from the R environment (R Core Team, 2020).

### 2.4 Statistical analysis

#### 2.4.1 Distress calls

Each individual was characterized by the mean value of every acoustic variable of its calls. Those individuals that emitted harmonic and noisy calls were characterized independently for both call types. Preliminary *t*‐tests did not show differences between sexes, and therefore, this variable was not considered in the following analyses. We compared populations using a Chi‐square test for the proportions of calls with ultrasonic components and *t*‐tests for the rest of the acoustic variables and body sizes. We also applied ANCOVAs to evaluate differences in the spectro‐temporal variables between populations while controlling for body size. To determine whether individuals that emitted harmonic calls could be grouped according to their origin, we applied a Stepwise Discriminant Analysis followed by a Canonical Analysis. We obtained the discriminant functions and determined the original variables with the highest correlation with these functions. Analyses were performed using Statistica V11^®^.

#### 2.4.2 Tympanic sensitivities

Statistical analyses were performed using the R environment (R Core Team, 2020). Preliminary ANOVA tests showed significant differences in body sizes between populations (including all the individuals of each population *F*_(1, 20)_ = 10.69, *p* = .004; including all the individuals from the south and a reduced subsample of eight individuals from the center *F*_(1, 15)_ = 10.59, *p* = .005). There were, however, no differences between sexes (total *F*_(1, 20)_ = 0.016, *p* = .90; subsample *F*_(1, 15)_ = 0.03, *p* = .88). Therefore, body size, but not sex, was included in the following analyses. The effects of body size (SVL), stimulus level (SPL), and population (POP) on the tympanic responses were evaluated fitting linear mixed‐effects models using the “lme4” package (Bates et al., 2015). These factors and the interaction between SPL and POP were included as fixed effects. The spectral cross‐correlations were run independently for each population, and models included the effects of SVL, SPL, call origin (CO; local vs. non‐local), and the interaction between SPL and CO. In all models, individual intercepts were included as a random effect to account for data dependence. Backward elimination of fixed effects and the significance of the terms included in the final model were obtained using the Satterthwaite's degrees of freedom method and a type III ANOVA using the “lmerTest” package (Kuznetsova et al., 2017). Finally, when SPL and PO (or CO) were significant, and pairwise differences of least square means were obtained to determine differences between populations (or call origin) at a given SPL. We implemented planned contrasts using the “emmeans” package, including a multivariate‐*t* adjustment for *p*‐values (Lenth, 2020). Outlier data were removed using the “rmor.fnc” function from the “LMERConvenienceFunctions” package, which excludes data with a standardized residual distance greater than 2.5 of the standard deviations (Tremblay & Ransijn, 2015). We used the package “bestNormalize” (Peterson & Cavanaugh, 2020) to improve the normality of the response variables.

## 3 RESULTS

### 3.1 Distress calls

Table 1 shows the call emission by population. Most of the central lizards (88%, *n* = 12) emitted distress calls (i.e., two females did not vocalize), while all the southern lizards (*n* = 26) did so. On average, however, each central lizard vocalized more than southern lizards (Table 1). All calls from the central population were harmonic, while most southern calls were noisy (66.3%; Table 1). Most harmonic calls were simple: 58.5% and 75%, for the central and southern populations, respectively (Table 1).

**TABLE 1:** Mean ± standard error (range) of the total number of distress calls emitted by individuals from the central (Isla de Maipo) and southern (Pucón) populations of *Liolaemus chiliensis*

Type of calls	*N*	Central population *n* = 12	*N*	Southern population *n* = 26	*t*_36_ (*p*‐value)
Total	171	14.25 ± 2.61 (2–33)	190	7.31 ± 1.37 (1–24)	**2.62 (<.05)**
1‐Harmonic	171	14.25 ± 2.61 (2–33)	64	2.46 ± 0.46 (0–9)	**5.87 (**≪**.001)**
Simple	100	8.33 ± 1.29 (1–15)	48	1.85 ± 0.38 (0–7)	**5.43 (**≪**.001)**
Complex	71	5.92 ± 1.92 (0–25)	16	0.62 ± 018 (0–3)	**4.43 (**≪**.001)**
2‐ Noisy	0	–	126	4.85 ± 1.40 (0–22)	–

Note

*n* = number of individuals included in the analysis, *N* = total number of calls recorded. *t*‐test (*p*‐values) = inter‐population comparisons of occurrence of the different call types; significant results (*p* < .05) are in bold.

Based on the frequency‐modulation patterns, we found five types of simple calls (see Labra et al., 2013), and their relative occurrence for the central and southern population, respectively, were: downward (40%, 60.5%), invariant (27%, 8.3%), upward (16%, 10.4%), bell‐shaped (13%, 8.3%), and U‐shaped (4%, 12.5%).

While 41.5% of the harmonic calls from the central population were complex, that is, they exhibited nonlinear phenomena, only 25% of the southern calls did so. We found three types of nonlinear phenomena (Figure 2a; Fitch et al., 2002; Labra et al., 2013), and their relative occurrence for the central and southern population were, respectively: deterministic chaos (40.8%, 50%), frequency jumps (18.3%, 12.5%), and sub‐harmonics (4.2%, 12.5%). Some calls had more than one type of these phenomena (Figure 2a; 28.2%, 18.75%), while others had a silence interval instead of any nonlinearity (8.5%, 6.25%).

**FIGURE 3:**
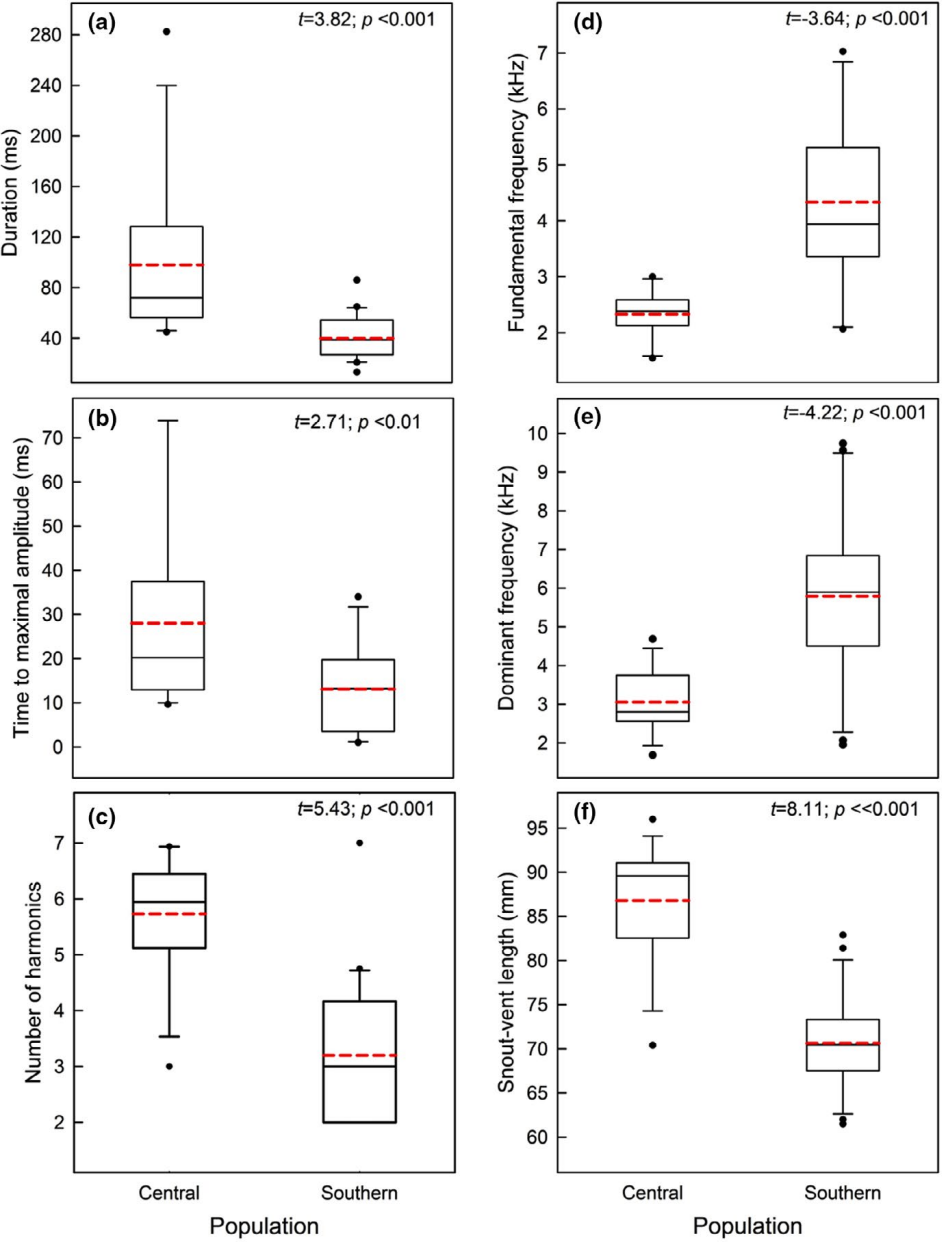
Box plots of the five acoustic variables of the harmonic calls and the snout‐vent length (SVL) of both populations of *Liolaemus chiliensis* (sample sizes for the central and southern populations are, for acoustic variables 12 and 21, and for the SVL 14 and 26). Boxes correspond to first and third quartiles and horizontal lines inside boxes to second quartiles (medians). Vertical lines correspond to error bars, black dots are outliers, and the red dashed lines are the mean values. Each plot shows the result of the interpopulation comparison (*t*‐test and *p*‐value). The degrees of freedom of the tests for the acoustic variables (a, b, c, d, and e) were 31 and 38 for the SVL comparison (f)

Considering together harmonic and noisy calls, a higher percentage of the central calls exhibited ultrasonic components, compared to the southern calls (26.9% vs. 1.05%, respectively; *χ* ^2^ = 52.16; *p* < .0001). Populations differed significantly in all the spectro‐temporal variables of their harmonic calls (Figure 3); those from the central population lasted longer (Figure 3a), took longer to reach the maximum amplitude (Figure 3b), had more harmonics (Figure 3c), and lower fundamental and dominant frequencies (Figure 3d,e) than southern calls. The mean duration of the noisy calls emitted by 14 southern lizards was 47 ± 3.8 ms, which was similar to the duration of the harmonic calls of this population (*t*_13_ = 1.20; *p* = .24). Finally, the mean call level of the southern population was 46.0 ± 0.33 dB SPL (*n* = 9 individuals), a value significantly lower (*p* < .001) than the one reported for a population geographically close to the studied central population (Melipilla: 62.53 ± 0.31 dB SPL; Labra et al., 2013).

The discriminant analysis of the harmonic calls showed differences between populations (Wilks’ *λ* = 0.41, *F*_(3, 29)_ = 14.1, *p* < .00001), and all the variation was explained by only one discriminant function (Table 2). This included call duration, dominant frequency, and the number of harmonics, and this last variable was the most relevant for discriminating the populations (Table 2). Stepwise discriminant analysis showed that 91.0% of individual calls were classified correctly according to their origin. The misclassified calls were from two individuals (out of 12) from the center and one (out of 21) from the south.

**TABLE 2:** Stepwise Discriminant Analysis for the acoustic characteristics of the harmonic distress calls of two populations of *Liolaemus chiliensis*

Variable	Discriminant function 1
No. harmonics	−0.587
Dominant frequency	0.484
Call duration	−0.394
Eigenvalue	1.462
Proportion explained variation	1.000

Note

The table shows the discriminant function scores obtained, the eigenvalue, and the proportion of the explained variance by the discriminant function of the three variables that the model included, which allowed separating calls (i.e., individuals) of both populations.

Populations differed in body size, and central lizards were larger than the southern ones (Figure 3f). After controlling for body size, there were no population differences in any of the studied variables (ANCOVAs; *p* > .05).

### 3.2 Tympanic sensitivities

**FIGURE 4:**
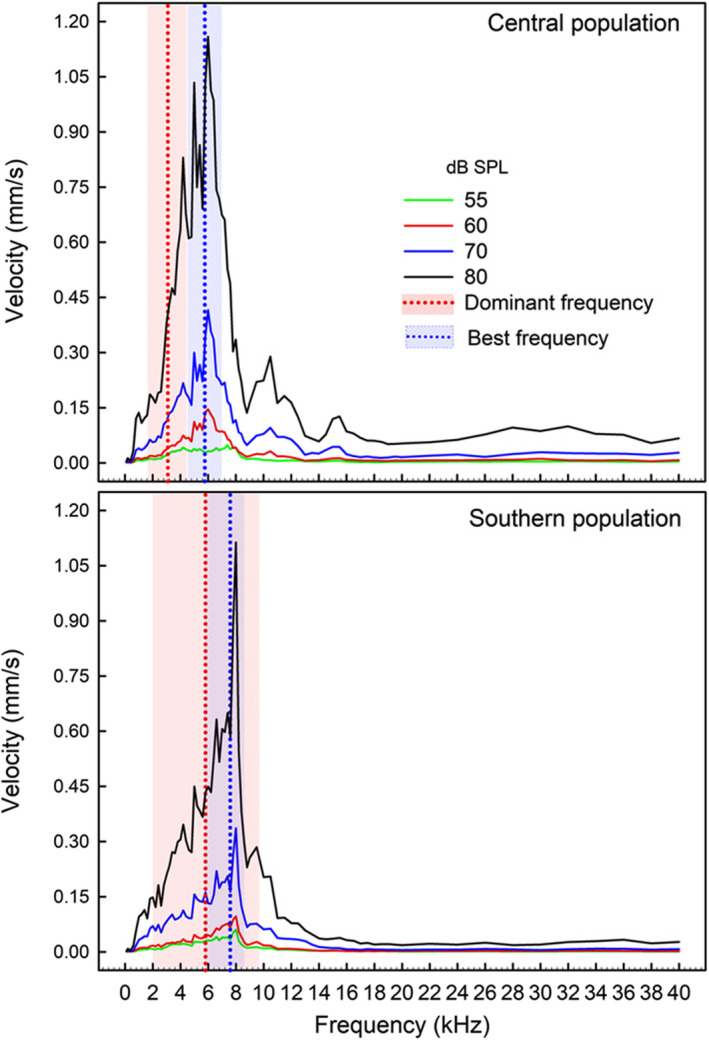
Tympanic velocity in responses to tones of different frequencies (100 Hz to 40 kHz) for the central (Isla de Maipo) and southern population (Pucón) of *Liolaemus chiliensis*. Responses to tones at four stimulus levels (55, 60, 70, 80 dB SPL) are depicted by continuous lines of different colors. The red vertical dotted line indicates the mean dominant frequency of the harmonic calls, and the shaded red area encompasses the range between the minimum and the maximum values recorded. The blue vertical dotted line indicates the mean best frequency using the data from the four stimulus levels, and the shaded blue area includes the sensitivity range (see Material and Methods for detailed explanations)

**FIGURE 5:**
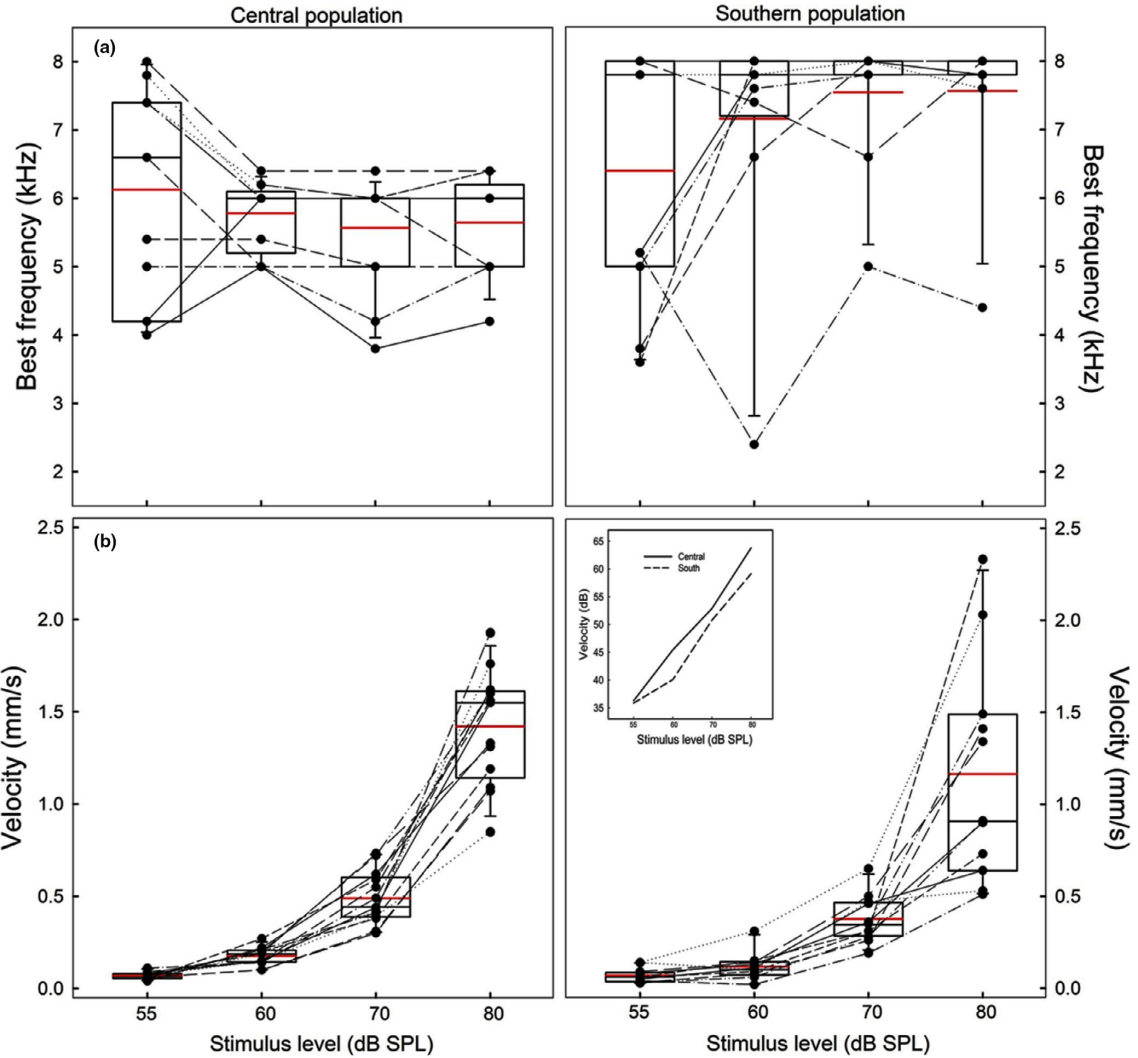
Box plots of two tympanic responses, the best frequency (a, top panels) and velocity (b, bottom panels) obtained with pure tones at four stimulus levels (55, 60, 70, 80 dB SPL) for the central (Isla de Maipo, left side) and southern population (Pucón, right side) of *Liolaemus chiliensis*. Boxes correspond to first and third quartiles and horizontal lines inside boxes to second quartiles (medians). Vertical lines correspond to error bars, and the red lines are the mean values. Black dots and thin lines between boxes represent data of individual subjects; outliers are included. Insert: Median values of the tympanic velocity (dB) of the central and southern populations

**FIGURE 6:**
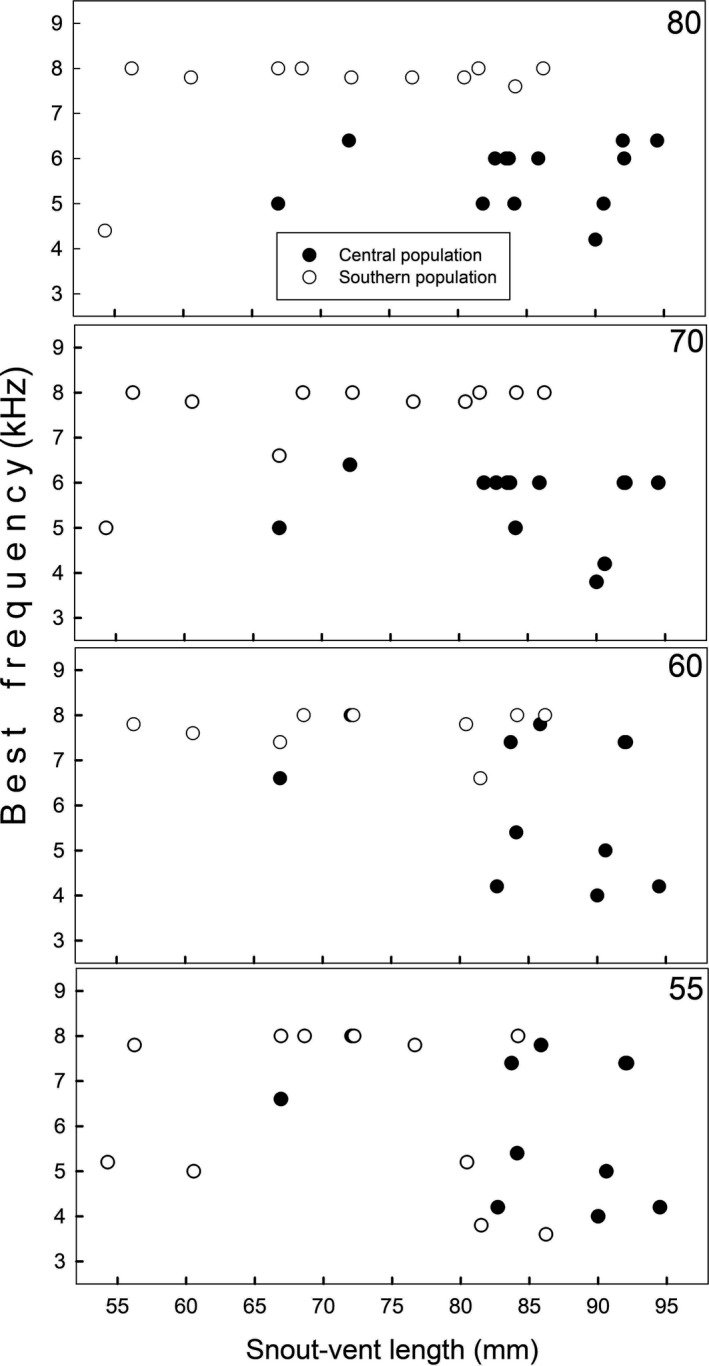
The mean best frequency (kHz) of each individual of both populations of *Liolaemus chiliensis* recorded at four stimulus levels (55, 60, 70, 80 dB SPL) as a function of their snout‐vent length (mm)

Figure 4 shows the tympanic responses of both populations obtained at four stimulus levels, between 0.1 and 40 kHz; there was no response above 12–14 kHz. The best frequency to pure tones differed between populations (POP; Table 3); overall, the southern population showed higher values than the central population (Figures 4 and 5a). Body size did not modulate this or any tympanic response, and Figure 6 shows, for example, that at similar body size, the southern lizards usually had higher best frequencies than individuals from the central population.

**TABLE 3:** Results of the Linear Mixed‐Effects Models to determine the effects of the population (POP), stimulus level (SPL), call origin (CO, local vs. non‐local), and snout‐vent length (SVL) on tympanic responses

Variable	Factor	Degree of freedom		*F*	*p*
		Numerator	Denominator		
Best frequency	POP	1	21.65	23.08	<.001
Velocity	POP	1	22.05	5.17	.033
	SPL	3	63.76	567.03	<.001
Min frequency	–	–	–	–	–
Max frequency	POP	1	21.68	25.90	<.001
	SPL	3	59.10	10.46	<.001
	POP: SPL	3	59.10	14.57	<.001
Frequency range	SPL	3	64.80	3.50	.020
Central‐Pop.	CO	1	52.00	8.24	<.001
Cross‐	SPL	3	46.11	42.77	<.001
Correlation					
Southern‐Pop.	CO	1	69.13	4.47	.038
Cross‐	SPL	3	68.97	32.01	<.001
Correlation					

Note

The variables analyzed were the best frequency, the velocity at the best frequency, sensitivity range (i.e., the frequency range over which the eardrum vibrated at least at half of the velocity recorded at the best frequency), the lower and upper frequency limits of this range, and the spectral cross‐correlations of each population. *F* indicates the value of the *F*‐statistic and *p* the probability.

The sound level (SPL) and population (POP) modulated the velocity transfer function at the best frequency (Table 3); in both populations, the velocity increased with the sound level, and higher values were recorded in the central population (Figure 5b). Paired comparisons between populations at a given SPL differed significantly, and the central population always showed higher velocity values than the southern population (Figure 5b; see Supplementary Material 1). The median values of the velocity in dB (re 1 μm/s) of each population (Figure 5b insert) show an overall linear tympanic response.

**FIGURE 7:**
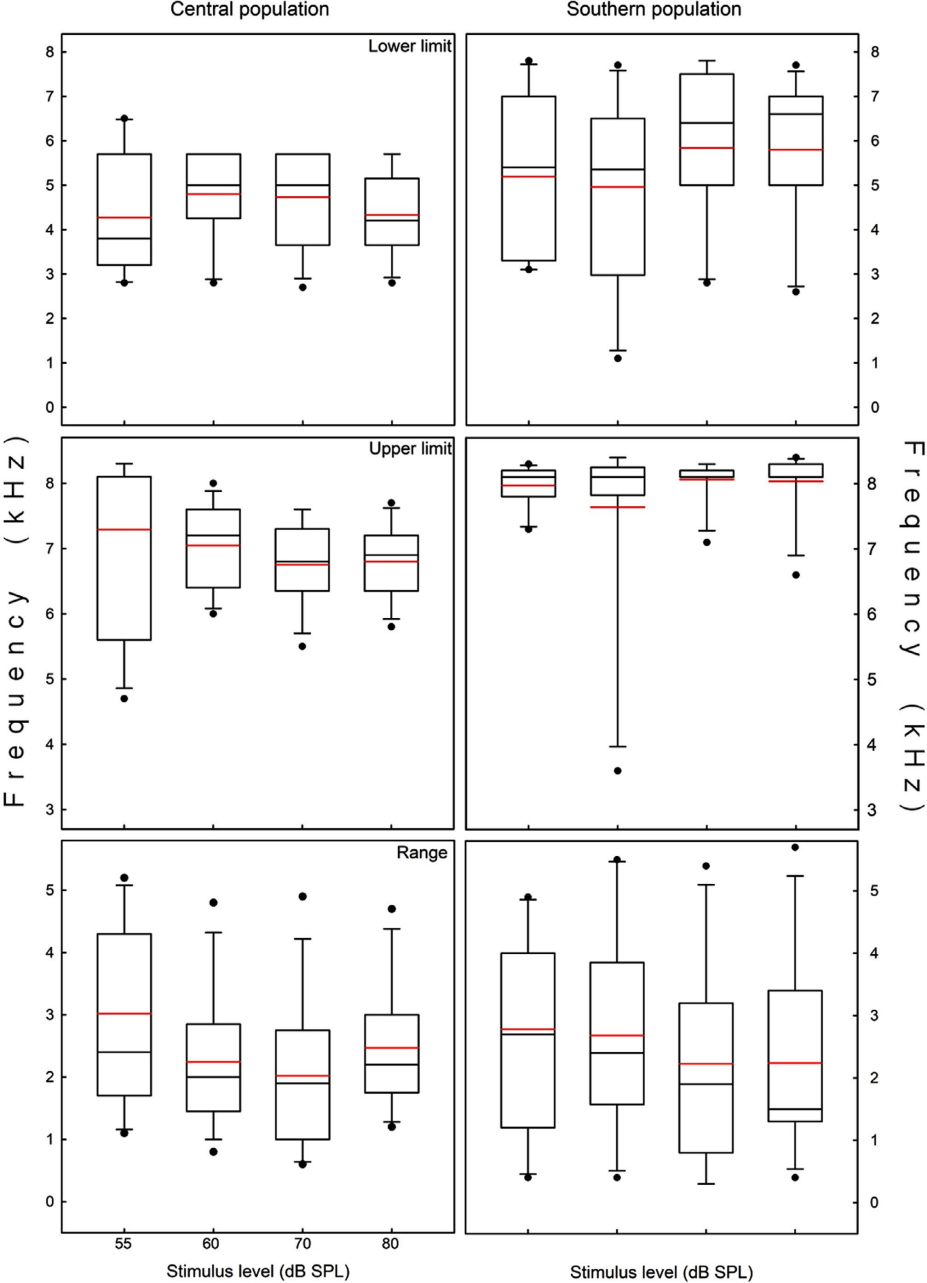
Box plots of tympanic sensitivities recorded for the central population (Isla de Maipo) and southern population (Pucón) of *Liolaemus chiliensis* at four stimulus levels (55, 60, 70, 80 dB SPL). The top and middle panels show the lower and upper frequency limits of the sensitivity range, and the bottom panels show the frequency range at which the eardrum vibrated at least at half of the velocity recorded at the best frequency. Boxes correspond to first and third quartiles, and horizontal lines inside boxes are the second quartiles (medians). Vertical lines correspond to error bars, black dots are outliers, and red lines are mean values

The lower frequency limits of the sensitivity range were unaffected by the factors studied (Table 3; Figure 7). In contrast, population (POP), stimulus level (SPL), and their interaction modulated the upper frequency limit (Table 3), as the central population had significantly lower values than the southern population at 60, 70, and 80 dB SPL (see Supplementary Material 1). The central population had a significantly lower upper frequency limit at 55 dB as compared to the other stimulus levels; in contrast, the southern population had similar values at all the stimulus levels (Figure 7). Finally, the sensitivity range (i.e., the difference between the limits) was only affected by the stimulus level (Figure 7), but the single difference was a broader range at 55 than at 70 dB SPL.

### 3.3 Matching between call characteristics and tympanic sensitivities

**FIGURE 8:**
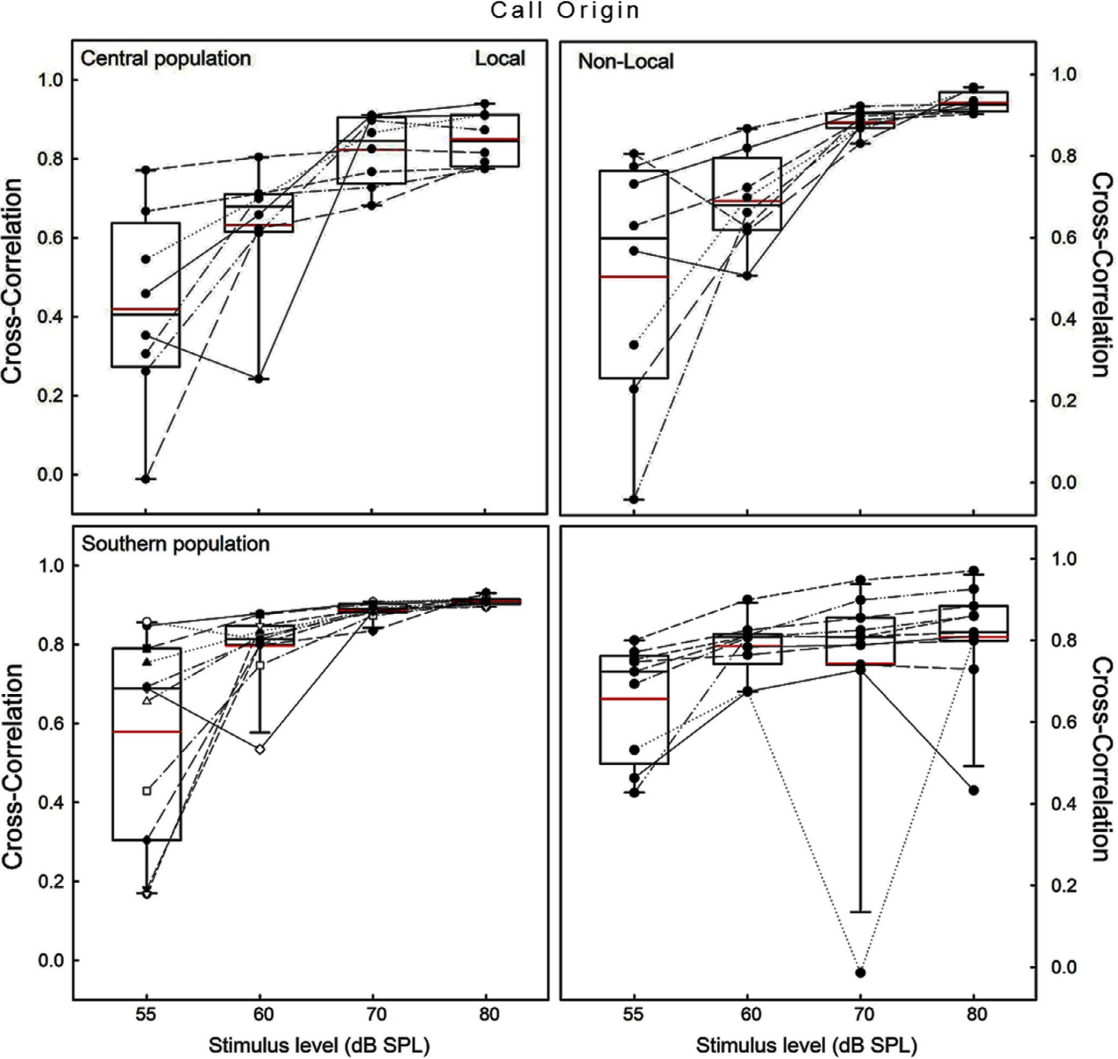
Box plots of the cross‐correlations between the spectra of the tympanic membrane and the synthetic distress calls of the central (top panels) and southern population (bottom panels) of *Liolaemus chiliensis*, exposed to calls of different origin: local (own) and non‐local (different) population. Boxes correspond to first and third quartiles, and horizontal lines inside boxes are the second quartiles (medians). Vertical lines correspond to error bars, and the red lines are the mean values. Black dots and thin lines between boxes represent data of individual subjects; outliers are included

For both populations, the spectral cross‐correlations between synthetic distress calls and tympanic sensitivities were modulated by call origin (CO) and stimulus level (SPL; Table 3, Figure 8). The cross‐correlations increased with stimulus level, and overall, the central population showed lower values for the local call than with non‐local call (i.e., southern). In contrast, the southern population had higher values for the local call. Paired comparisons at a given SPL value were significant (see Supplementary Material 1), and both populations showed better matches with the southern call (Figure 8).

Considering a mean best frequency to pure tones, pooling data from the four stimulus levels, of 5.78 and 7.17 kHz (Figure 4) for the central and southern populations, respectively, and that the corresponding mean dominant frequencies of the distress calls of these populations were 3.1 and 5.8 kHz (Figures 3 and 4), no population showed a strict match between the call characteristics and tympanic sensitivities. However, the southern population showed a slightly better matching than the central population (i.e., frequency differences: 1.37 vs. 2.68 kHz). In addition, in the southern population, the upper and lower limits of the sensitivity range encompass the mean and the range of the dominant frequency of its distress calls, which was not the case for the central population ranges (Figure 4).

## 4 DISCUSSION

The Weeping lizard populations differed in the signal and receptor characteristics, and yet only the southern population showed a high spectral cross‐correlation between the tympanic sensitivity and the local call. In addition, this population showed a relatively good match between the characteristics of its calls (i.e., dominant frequency) and the frequencies at which the tympanic membrane vibrated at the highest velocity (i.e., best frequency).

Below we analyze the results of each communication component and discuss the hypothesis that the extant differences in audio–vocal matching between populations have arisen during the evolutionary processes of acoustic communication in this lizard.

### 4.1 Distress calls

The population differences in the distress call characteristics support the notion of geographic variability in calls, previously proposed for this species (Labra et al., 2016; Pincheira‐Donoso & Núñez, 2005). These differences, however, disappeared when variables were corrected for body size; the southern population, with smaller body sizes, had calls with shorter duration, time to the maximum amplitude, and higher fundamental and dominant frequencies than the central population. The negative relationship between call frequencies and body size is similar to previous reports for different types of vocalization across taxa (Birds: Friis et al., 2021; Martin et al., 2011; Ryan & Brenowitz, 1985; Amphibians: Gingras et al., 2013; Tonini et al., 2020; Wilczynski et al., 1993; Crocodiles: Vergne et al., 2012; Mammals: Bowling et al., 2017; Newar & Bowman, 2020; Geckos: Rohtla Jr et al., 2019). It is not surprising this negative relation, considering that typically, larger animals have larger structures that result in the production of lower frequencies (Fletcher, 2004; Bowling et al., 2017; see Riondato et al., 2021 for an exception).

Noisy calls, not reported previously for this lizard species (Labra et al., 2013), were only emitted by the southern population, where these predominated. This dissimilarity in the emission of noisy calls may also be a consequence of the population difference in body size. Gingras et al. (2013), in a comparative study on anurans, showed that larger species emitted more harmonic calls, proposing that this may be a consequence of a more developed vocal structure (Rohtla Jr et al., 2019; Russell et al., 2014). The difference in body size between populations may determine a differential development of the vocal apparatus, and the southern population potentially has a less developed structure. This variation may also explain the difference in the number of harmonics found in the harmonic calls. In humans, for example, lesser development of vocal apparatus results in vocalizations with fewer harmonics (Godoy et al., 2020).

The southern lizards also emitted fewer distress calls, which may be related to less predation risk, much as bird species with low predation risk emit fewer distress calls (Møller & Nielsen, 2010). Predation pressures experienced by populations of the Weeping lizard are not known, although based on data for other *Liolaemus* species, the southern population may have less predation pressure, at least from “non‐traditional” lizard predators (e.g., spiders, Reyes‐Olivares et al., 2020; passerine birds, Troncoso‐Palacios et al., 2020), because this kind of threat has been only reported to affect lizards in the northern and central portions of the Weeping lizard distribution. Furthermore, fewer calls of the southern population have ultrasonic components, which are likely to encode messages for predators rather than for conspecifics (see next section; Labra et al., 2013). As such, comparatively low predation pressure, including that from predators sensitive to high frequencies (e.g., especially mammals), may contribute to reduce the rate of distress call emission in southern populations of this lizard.

The emission rate of distress calls may also be affected by body size; Forti et al. (2018), in a comparative study of the evolution of anuran distress calls, found that the smaller species tend to lack distress calls. The authors proposed that a small body size might constrain the emission of enough long and/or loud calls to be effective. The southern calls are shorter and weaker than those emitted by a central population (Labra et al., 2013; present study). In addition, these calls contain high frequencies, which attenuate faster as they propagate (Bradbury & Vehrencamp, 2011), reducing their efficiency in long‐range communication. In summary, the potential low effectiveness of the southern calls and/or a reduced predation risk may explain the comparative lower call production of this population.

The selective pressures involved in the inter‐population variation in body size of this lizard species remain unclear. Different environmental factors can modulate animal body sizes (Amado et al., 2019), although temperature seems to be a relevant factor for various taxa (Velasco et al., 2020). A thermal decrease with latitude occurs in Chile (di Castri & Hajek, 1976), and the lower southern temperatures may select for smaller body sizes, following the reverse Bergmann's rule, as most Squamata do (Ashton & Feldman, 2003; Oufiero et al., 2011; but see Velasco et al., 2020).

### 4.2 Tympanic sensitivities

The Weeping lizard has sensitivity frequency ranges (i.e., the range over which the eardrum vibrated at least at half of the velocity recorded at the best frequency) of 4.5–7.0 kHz and 5.4–7.9 kHz for the central and southern populations, respectively. These values are within the hearing ranges for different lizard species (0.1–8 kHz; Wever, 1978; Manley, 2000, 2011), although a few species have high‐frequency hearing (up to 14 kHz; Christensen‐Dalsgaard & Manley, 2005, 2008; Manley & Kraus, 2010). In the Weeping lizard, no tympanic response was recorded above 12–14 kHz, suggesting that frequencies above 12 kHz do not encode information for conspecifics. Different gekkonid lizards have vocal spectra with frequencies not detected by their auditory receptors (e.g., Brown, 1985; Manley & Kraus, 2010; Werner & Wever, 1972), which led to propose that these frequencies are relevant for predators (Brown, 1985; Frankenberg, 1975; Rohtla Jr et al., 2019; Russell et al., 2014); potentially, this may also be the case for the Weeping lizard (Labra et al., 2013).

The population differences in best frequency (i.e., the frequency at which the eardrum vibrates at the maximum velocity) were unrelated to the body size differences; at equal size, southern lizards usually had higher best frequencies. Similarly, Werner and Igic (2002) reported that the best frequencies of different gekkonids did not correlate with body size. These results, however, contrast with data for some frog species showing a negative association between the best frequencies and body size (Keddy‐Hector et al., 1992; Wilczynski & Ryan, 1999). Therefore, it is likely that hearing of the Weeping lizard is influenced by particular physical and/or ecological constraints, which would mask the body‐size effects.

The mean tympanic velocities measured at the best frequencies ranged from 0.07 mm/s at 55 dB SPL for both populations to 1.42 and 1.17 mm/s at 80 dB SPL for the central and southern population, respectively. These values are within the lower limit of those reported for other lizard species at different stimulus levels (e.g., between 0.2 and 6.9 mm/s; dB SPL: 100, Saunders et al., 2000; Werner et al., 2002; dB SPL: 94, Christensen‐Dalsgaard & Manley, 2005; dB SPL: 70, Han & Young, 2018). We cannot rule out, however, that higher velocities could have been recorded for these populations if the laser beam had been aimed at locations other than the insertion point of the extracolumella, considering that the eardrum stiffness varies across its surface (Han & Young, 2018; Werner et al., 1998) and that the eardrum exhibits frequency‐dependent vibration modes (Bergevin et al., 2015).

The eardrum of the central lizards vibrated at higher velocities than the southern lizards, which may reflect population differences in the eardrum properties (e.g., mass, stiffness; Saunders et al., 2000). We previously suggested that the studied populations may be subjected to physical and/or ecological constraints that may mask the effects of body size. This is also supported when it is considered that in other lizard species, the maximum tympanic membrane velocity correlates with body size (Werner et al., 2002, 2008). Presently, however, it remains unclear which factors modulate or constrain hearing in the Weeping lizard.

### 4.3 Matching between call and tympanic characteristics

The matching between call characteristics and auditory sensitivities rarely had been explored in lizards, but the few cases in gekkonids show a match between both domains (Brittan‐Powell et al., 2010; Chen et al., 2016; Manley & Kraus, 2010). Of the studied populations, only the southern one had a relatively good match between the communication components and showed a high spectral cross‐correlation between the tympanic response and the local distress call. In the central population, the range of the dominant frequencies did not overlap with the sensitivity range of the best frequencies, and this population showed a better spectral cross‐correlation with the non‐local distress call. This last result seems contradictory with data from the population of Melipilla, located close to our central population of Isla de Maipo, in which lizards only responded behaviorally to the local call (Labra et al., 2016). Potentially, call characteristics may show a better matching at central levels of the auditory system, as in some lizard species there is variation in the highest sensitivities recorded in the middle ear, inner ear, and the auditory neurons at central levels (Brittan‐Powell et al., 2010; Manley, 2000).

The differences in matching in the communication components between the Weeping lizard's populations may relate to the evolution of distress calls along with the historical dispersal processes of this species. The Zero‐Force Evolutionary Law proposes that in any evolutionary system, diversity and complexity tend to increase (McShea & Brandon, 2010). In this scenario, the radiation center of this species would be the south, where the novelty, a simple and short distress call, evolved. From here, an expansion toward the north may have involved new selective pressures (e.g., higher environmental temperature, predation risk), promoting, among other features, an increase in body size with concomitant changes in the size of vocal structures, and in the distress call characteristics, resulting in more complex calls. In this scenario, the southern population may have been exposed for a longer period to selection pressures promoting a better match between the signal and its detection. Changes in the middle ear would take longer than those affecting vocalizations, originating the mismatch between the communication components observed in the central population; this also might explain the high spectral cross‐correlation of the tympanic sensitivity with the non‐local distress call. Faster evolution of signals than the reception/recognition system was proposed by Betancourth‐Cundar et al. (2016) for the frog *Allobates femoralis*; the authors found a decoupled evolution between signals (i.e., advertisement calls) and male–male recognition across different populations, suggesting that signal recognition evolves slower than call changes. In the same line, Penna et al. (2015) proposed that the diversification process of the *Alytes* frogs may cause a secondary mismatch between call frequencies and the auditory sensitivity recorded in some species.

The alternative scenario, an initial evolution of a complex distress call at the central population with a secondary reduction of this complexity associated with the colonization of the southern areas, seems less plausible (McShea & Brandon, 2010). This scenario requires the occurrence of vocalizations in the *Liolaemus* ancestor, and thus, the Weeping lizard might have initially evolved a highly developed vocalization with the associated vocal apparatus. Subsequently, a secondary reduction of the distress call expression and complexity would have occurred along with a southern expansion. This scenario, however, is not well supported, as the Weeping lizard is the only species in this genus that vocalizes (Reyes‐Olivares & Labra, 2017). In addition, this scenario does not provide parsimonious explanations for the better tympanic response to a non‐local call in the central population.

This study explored variation in acoustic signals and tympanic sensitivity in the Weeping lizard, considering conspecifics as the target of the distress calls (e.g., Ruiz‐Monachesi & Labra, 2020). However, this evolutionary novelty may have predators as the main target rather than conspecifics, which may account for the observed mismatch in the communication components of this lizard species. As mentioned, the actual predators of this lizard have not been identified, and thus, we cannot relate the characteristics of its distress calls and/or hearing sensitivities with the vocalization characteristics and hearing abilities of particular predators. However, the guild of vertebrate predators described for central Chile (i.e., carnivores, raptors, snakes; Jaksić et al., 1981) would be the same for both populations (Iriarte & Jaksić, 2017; Iriarte et al., 2019). Thus, even considering that the southern population may have a lower predation risk, as we discussed above, if distress calls are directed to predators, either to startle the primary one (Neudorf & Sealy, 2002) or to attract secondary predators (Högstedt, 1983; Schuett & Gillingham, 1990), call similarities between the two populations would have been expected, which is not the case. As for the possibility that the tympanic sensitivities of this lizard evolved to respond to the predator vocalizations, based on the present results, this lizard species may react to vocalizations of raptors (e.g., range of frequencies 0.6–10 kHz; Jurisevic, 1998) and/or of canids, such as *Lycalopex culpaeus* (Cohen & Fox, 1976). However, considering that the vocalizations of at least one of this lizard's predators, *Geranoaetus (Buteo) polyosoma* (Jaksić et al., 1981), do not show geographic variation (Farquhar, 1998), it would be expected that both lizard populations show similarities in their hearing sensitivities, which is not the case. Some of these lizard predators, however, show geographic variation in body size, following Bergmann's rule (Jiménez et al., 1995), which may determine differences in the frequencies of their vocalizations (e.g., Bowling et al., 2017; Friis et al., 2021; Martin et al., 2011; Wilczynski et al., 1993). Therefore, for example, predator populations from the central region may have vocalizations with higher frequencies, which would be less likely to be detected by the central population of the Weeping lizard. In summary, indirect evidence does not support the hypothesis that the evolution of the studied communication components has been determined by the direct interaction with predators. Nevertheless, it will be necessary to identify the main predators of this lizard species, as well as, to test the ability of this lizard to respond to predator vocalizations and the responses of the main predators to the lizard's distress calls, considering separately central and southern populations.

## 5 CONCLUSIONS

Evolutionary novelties allow organisms to develop new functions within new ecological niches (Pigliucci, 2008), and the distress calls of the Weeping lizard provide information on the predation risk to conspecifics (Hoare & Labra, 2013; Labra et al., 2016; Ruiz‐Monachesi & Labra, 2020), but not to a congeneric and syntopic species (Fong et al., 2021). However, even though this evolutionary novelty is present in both studied populations (Labra et al., 2016), they differ in the matching between signal and receiver characteristics. This suggests that different evolutionary histories and/or selective pressures have affected these populations. In addition, the vocal and auditory components seem to differ in the selective pressures, since call structure depends on body size, while tympanic sensitivity seems not to be affected by this factor in the study species. Thus, the matching of the communication components of this novelty may not be tightly associated with strong selective pressures that ensure the coevolution of its components. A phylogeographic analysis of this lizard species, combined with comparative morphology of its vocal apparatus and middle and inner ears, is necessary for further insights into the evolution of its distress calls and auditory processing.

## CONFLICT OF INTEREST

The authors declare that they have no conflict of interests.

## AUTHOR CONTRIBUTION

**Antonieta Labra:** Conceptualization (lead); Data curation (lead); Formal analysis (equal); Funding acquisition (lead); Investigation (lead); Methodology (equal); Project administration (lead); Resources (lead); Software (supporting); Supervision (lead); Validation (lead); Visualization (lead); Writing – original draft (lead); Writing – review & editing (lead). **Claudio Reyes‐Olivares:** Conceptualization (supporting); Data curation (supporting); Formal analysis (equal); Investigation (equal); Methodology (equal); Visualization (supporting); Writing – original draft (supporting); Writing – review & editing (supporting). **Felipe N. Moreno‐Gómez:** Data curation (equal); Formal analysis (lead); Methodology (lead); Software (lead); Visualization (supporting); Writing – original draft (supporting); Writing – review & editing (supporting). **Nelson A. Velásquez:** Investigation (supporting); Methodology (supporting); Writing – review & editing (supporting). **Mario Penna:** Formal analysis (supporting); Methodology (supporting); Resources (supporting); Writing – review & editing (supporting). **Paul H. Delano:** Formal analysis (supporting); Methodology (supporting); Writing – review & editing (supporting). **Peter M. Narins:** Data curation (supporting); Supervision (supporting); Writing – review & editing (supporting).

## Supporting information

Supplementary Material

## Data Availability

Data will be available at https://doi.org/10.5061/dryad.mw6m905z2.
